# Diagnostic Accuracy of Ultrasound in the Diagnosis of Small Bowel Obstruction

**DOI:** 10.3390/diagnostics9030088

**Published:** 2019-08-06

**Authors:** Stefania Tamburrini, Marina Lugarà, Francesco Iaselli, Pietro Paolo Saturnino, Carlo Liguori, Roberto Carbone, Daniela Vecchione, Roberta Abete, Pasquale Tammaro, Ines Marano

**Affiliations:** 1 Department of Radiology, Ospedale del Mare-ASLNa1 Centro, 80147 Napoli, Italy; 2 Department of Internal Medicine, Ospedale del Mare-ASLNa1 Centro, 80147 Napoli, Italy; 3 Department of General Surgery, Ospedale del Mare-ASLNa1 Centro, 80147 Napoli, Italy

**Keywords:** abdominal ultrasound, bowel ultrasound, small bowel obstruction, point of care ultrasound, bedside ultrasound, emergency ultrasound

## Abstract

Introduction: Small bowel obstruction (SBO) is a common presentation to the Emergency Department (ED). This study aimed to analyze the accuracy of ultrasound (US) in diagnosing and staging SBO. Objectives: The main object of this study was to analyze the accuracy of ultrasound in diagnosing and staging SBO compared to CT. Methods: Retrospectively, stable patients with an ultrasonographic diagnosis of SBO who underwent abdominal CT immediately after US and before receiving naso-intestinal decompression, were included. US criteria for the diagnosis of SBO were related to morphological and functional findings. US diagnosis of obstruction was made if fluid-filled dilated small bowel loops were detected, peristalsis was abnormal and parietal abnormalities were present. Morphologic and functional sonographic findings were assigned to three categories: simple SBO, compensated SBO and decompensated SBO. US findings were compared with the results of CT examinations: Morphologic CT findings (divided into loop, vascular, mesenteric and peritoneal signs) allowed the classification of SBO in simple, decompensated and complicated. Results: US diagnostic accuracy rates in relation to CT results were calculated: ultrasound compared to CT imaging, had a sensitivity of 92.31% (95% CI, 74.87% to 99.05%) and a specificity of 94.12% (95% CI, 71.31% to 99.85%) in the diagnosis of SBO. Conclusions: This study, similarly to the existing literature, suggests that ultrasound is highly accurate in the diagnosis of SBO, and that the most valuable sonographic signs are the presence of dilated bowel loops ad abnormal peristalsis.

## 1. Introduction

Small bowel obstruction (SBO) refers to partial or complete blockage of the small intestine, generally with a sudden onset. SBO etiology in developed countries includes adhesions (74%), Crohn’s disease (7%), neoplasia (5%), hernia (2%), radiation (1%) and miscellaneous (11%). In contrast, developing countries, etiology includes adhesions (34%), hernia (16%), malignancy (13.5%) and tuberculous stricture (10%); acute intestinal obstruction due to foreign bodies is rare in adults. Previous abdominal surgery does not represent a dominant risk factor for SBO caused by solitary band adhesions, unlike SBO caused by matted adhesions; while in patients with no history of previous abdominal surgery (virgin abdomen) the risk for bowel obstruction is usually due to a solitary band [1,2]. The diagnosis of SBO in the Emergency Department (ED) has been estimated to be around 2% of all patients who presented with abdominal pain and 15% of all patients who ultimately get admitted to the surgical unit from the ED [3,4,5,6,7,8]. The clinical suspicion of SBO is usually made based on patient’s history, symptoms and physical sign (crampy abdominal pain, abdominal distension, nausea and vomiting). Delay in the diagnosis and management of SBO is associated with a higher risk of bowel resection, complications may be strangulation and bowel necrosis, and both may lead ultimately to perforation, sepsis and death [3,4,5,9,10]. The indications for and timing of surgical intervention for SBO have changed over the past decades. There is a widespread assumption that most of those conditions may resolve spontaneously if parietal vascular damage is absent with nonsurgical treatment, namely nasointestinal decompression (nasogastric tube insertion, bowel rest, intravenous fluids) [6,8,11]. The real dilemma that surgeons and radiologists face when confronted with a possible SBO is to confirm or exclude the pathology. Imaging should answer if the small bowel is obstructed or not, how severe the obstruction is, where it is located, what is its cause and if strangulation is present. Multimodality imaging (X-Rays, ultrasound, CT and MRI) has been proposed to confirm, stage and define the cause of SBO [12,13,14,15]. CT represents the gold standard imaging modality in the evaluation of SBO, answering to all diagnostic key points, in fact it can confirm the pathology, determining the cause and level of mechanical obstruction and the stage of the SBO, defining the presence or the absence of parietal damage; however, its use confers significant expense, potential delays and ionizing radiation. The use of point-of-care ultrasound (POCUS) for the evaluation of SBO has grown in recent years and ultrasound is increasingly being touted as a first-line imaging modality for SBO [6,15,16,17]. US usefulness was confirmed in several “formal” (radiologist-performed) studies. Moreover, point of care US at a patient’s bedside performed by an emergency physician has been proposed to confirm or exclude the presence of SBO in emergency setting [7,18,19,20,21,22]. The ability of POCUS to accurately diagnose SBO could potentially improve patient care by decreasing time to diagnosis and expediting consultation, as seen with other POCUS applications. Moreover POCUS can identify many other causes of abdominal pain (gallstones, abdominal aortic aneurysm, appendicitis, hydronephrosis suggestive of a kidney stone or intra- abdominal free fluid). In addition, ultrasound is likely to be the only imaging tool that is readily accessible in low resource settings, making it particularly useful when assessing a patient for a SBO when CT is either not available or prohibitively expensive [15].

Those studies demonstrated that there is not a significant difference in the accuracy of detecting SBO between ultrasound and CT [21]. Fluid-filled loops are easily visualized at US, and the differential diagnosis between a mechanical obstruction and paralytic ileus is made by visualizing peristaltic movement [23,24], but the underlying cause of ileus is found by ultrasound in only 45% of cases; meanwhile, CT can detect 100% of SBO causes [12,16,21,25,26]. Because a conservative approach of SBO is effective in avoiding the need for surgical intervention in approximately 65% of patients, ultrasound may rapidly redirect patients to conservative or surgical treatment. US in SBO should be “goal directed” to confirm or exclude the diagnosis. Moreover, repeated ultrasound examination in patients treated conservatively is safe and can be used to determine the progression or the resolution of obstruction [18] as part of diagnostic and follow-up process.

## 2. Materials and Methods

### 2.1. Objectives

The main object of this study was to analyze the accuracy of ultrasound in diagnosing and staging SBO compared to CT.

### 2.2. Patients

This was a retrospective, single-center cohort study in a public hospital. Patients presenting, between September 2018 and June 2019, with acute abdominal pain (crampy abdominal pain, abdominal distension, nausea and vomiting over a twelve month’s period), and who underwent abdominal/pelvic CT immediately after ultrasound examination before they received nasointestinal decompression, were included in the study. Two radiologists with 15 years of experience in Emergency Radiology reviewed ultrasound and CT examinations. This study was approved by the hospital’s Institutional Review Board (IRB) with waiver of informed consent.

### 2.3. Ultrasound Technique

Patients referred from the Emergency Department for abdominal ultrasound examination were scanned in the supine position without special preparation. Abdominal sonography was performed using a 3.5 MHz transducer to have a general overview of the abdomen; small bowel loops were searched in the central region of the abdomen and in the pelvis. In addition, a focused examination with a linear probe (7.5 MHz) was performed when patients referred a focal point of tenderness. Images and video were recorded for each patient.

All abdominal and pelvic quadrants were scanned to detect free fluid, and a systematic evaluation of the entire abdomen was conducted. Extra intestinal causes of abdominal pain were first excluded and comorbidities were annotated in the report. Ultrasound diagnosis of obstruction was made if fluid filled dilated small bowel loops were detected, peristalsis was abnormal and parietal abnormalities were present. Interference by gas echoes from distended bowel was avoided by scanning the distended abdomen using oblique or coronal planes, or gentle pression through moving the transducer slowly over the abdomen (graded compression) was applied to squeeze the air way from the region of interest. 

### 2.4. Ultrasound Diagnosis Criteria and Staging of Small Bowel Obstruction

**Figure 1:**
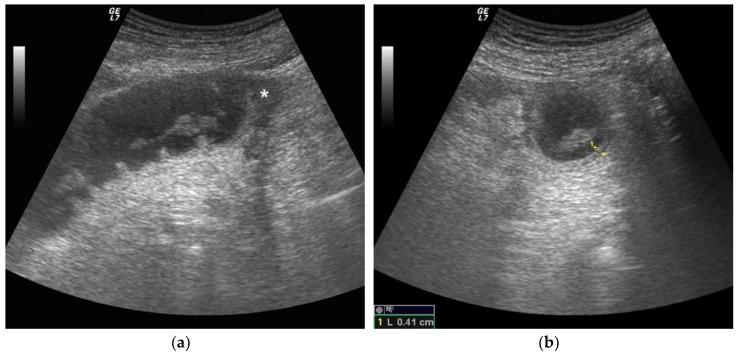
A 68-year-old female patient with previous abdominal surgery. Ultrasound image long (**a**) and axial (**b**) evaluation of a fluid-filled dilated small bowel loop with hyperechogenic floating material. Bowel peristalsis was absent. Mild parietal and valvulae conniventes thickening were present. Free fluid between bowel loops was detected (*). At surgery, mechanical obstruction due to a bridle was evident after release of adhesion, the bowel loop pinks up, peristalsis recovered and resection was avoided.

US criteria for the diagnosis of SBO were related to morphological and functional findings. Morphologic and functional sonographic findings were assigned to three categories: simple SBO, complicated SBO and decompensated SBO. Five findings were evaluated for the ultrasound diagnosis and staging of SBO: the presence of dilated loops, abnormal peristalsis, parietal and valvulae conniventes modification and the presence of free fluid. The first sonographic finding considered in this study was the presence or absence of dilated and fluid-filled loops related to an obstacle to the progression of intestinal contents and fluids (Figure 1a,b). Small bowel dilatation was defined as bowel diameter ≥25 mm measured from outer wall to outer wall [27]. 

**Figure 2:**
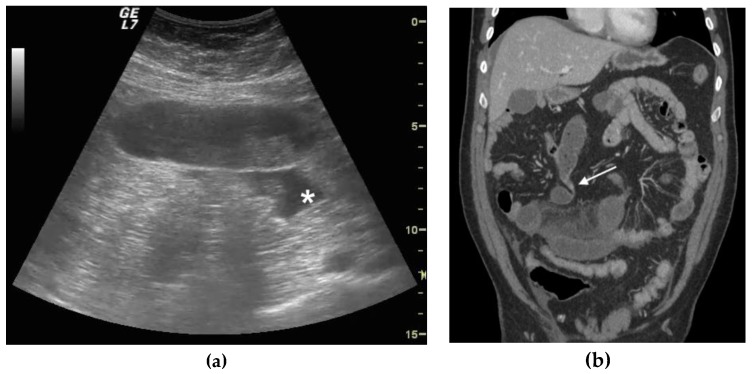
A 56-year-old male patient with virgin abdomen with compensated ileus. (**a**) Ultrasound long axis evaluation of a fluid-filled dilated small bowel loop, parietal was not thickened, free fluid was detected (*), peristalsis was ineffective with “back and forth” movements and defined decreased. (**b**) CT examination with IV contrast and coronal reconstruction. Small bowel fluid filled dilated loops were detected, and meso stranding and free fluid between bowel loops were also present. A focal point of stricture was evident (white arrow). At surgery, after release of adhesion, the bowel loop pinks up, peristalsis recovered and resection was avoided.

The second finding was determining and grading the presence of normal or altered peristalsis (hyperkinetic, decreased or absent). Ineffective peristalsis was defined as decreased in case of abnormal “back and forth” movements due to shuttling or swirling movements on intraluminal bowel contents. The third considered finding was normal or increased wall thickness, and given a lack of clear consensus on the maximum wall thickness in a normal bowel, no specific cut-off for bowel thickness was used. A non-thickened bowel layer is typical of a simple, non-complicated ileus. Wall thickening is usually detected in cases of vascular damage (complicated or decompensated ileus), mainly due to submucosal edema (Figure 1a,b). The fourth considered finding, always related to the vascular damage, was the increase in the thickness of the valvulae conniventes (Figure 2a,b) that project into the bowel lumen. The fifth considered finding was the presence or absence of free fluid between bowel loops. The presence of free peritoneal fluid is due to the bowel’s inability to resorb liquids and represents a sign of parietal damage; the bowel layers act as a sponge, determining the passage of fluid in the peritoneal cavity, and this finding is typical of complicated and decompensated ileus [28]. The above listed US findings have been attributed to one of the categories between simple ileus, complicated ileus and decompensated ileus [17,18,28,29,30]. (Table 1)

### 2.5. CT Criteria and SBO Staging 

CT was performed after the administration of intravenous contrast material, unless there were contraindications. The use of intravenous contrast was determined to assess for presence of parietal bowel ischemia or damage. In all cases, oral contrast medium was not administered. CT criteria for diagnosis for SBO, according to Paulson et al. [30], were considered major signs (small bowel dilated to 2.5 cm or greater) and the colon not dilated (<6 cm), the transition point from dilated to non-dilated small bowel, and minor (air fluid levels, colon decompressed) and minor ancillary findings included the “small bowel feces sign” just proximal to the transition point. CT stages of SBO vary depending on the involvement of loop, meso and peritoneal cavity and vascular parietal damage. In simple SBO, there is only loop involvement; in complicated SBO, loop and the peritoneal cavity are involved; and in decompensated, SBO loop and meso are involved and hypoattenuating bowel loops are present due to vascular parietal damage (Figure 2a,b) [31].

### 2.6. Data Analysis and Results

The demographics were characterized using descriptive statistics. The distributions of continuous data were examined and found to be slightly skewed. As a result, continuous variables were reported as medians with interquartile ranges. Standard 2 × 2 tables were used to calculate sensitivity, specificity and positive/negative likelihood ratio (+LR/−LR) with 95% confidence intervals (CI) for ultrasound compared to CT. Twenty-six patients with an ultrasound positive diagnosis of simple SBO who did not perform CT in the ER settings were excluded from the study.

Forty-three patients with clinical suspected diagnosis of bowel obstruction and who underwent ultrasound examination and CT immediately after, before nasointestinal decompression in ER setting, were included. The mean age was 63 y/o, and 60% of our patients were male and 40% female (26 M/17 F). 

**Figure 3:**
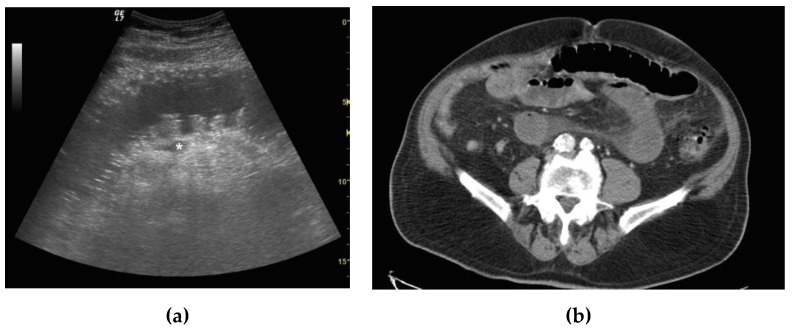

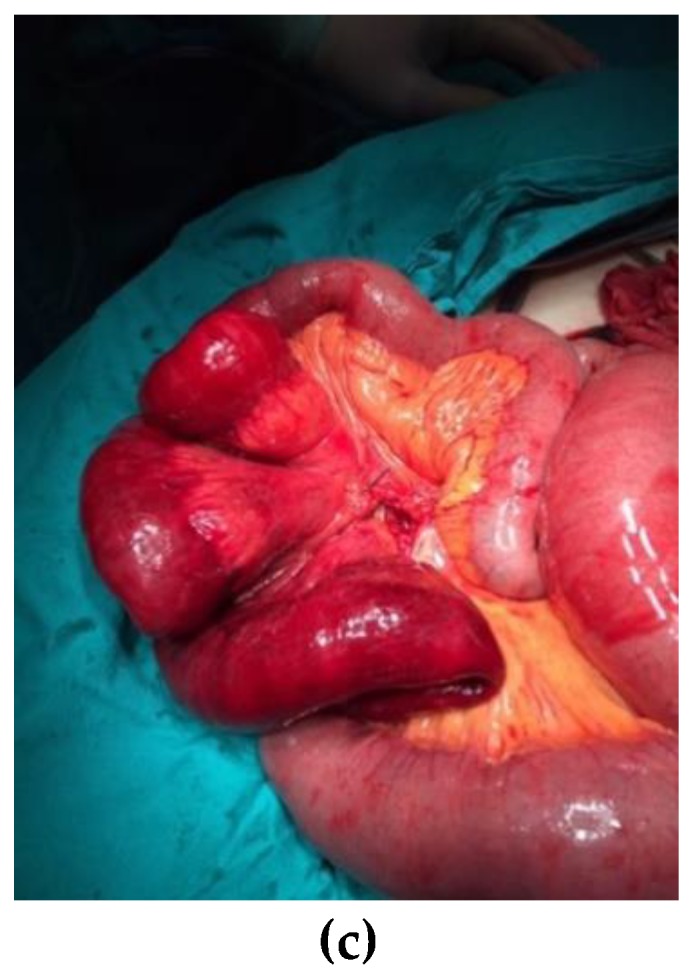
A 79-year-old male patient with previous abdominal surgery with decompensated ileus. (**a**) Ultrasound long axis evaluation of a fluid-filled dilated small bowel loop. Severe parietal and valvulae conniventes thickening were present, and fluid effusion adjacent was detected (*). Free fluid between bowel loops was also visualized. Peristalsis was absent. (**b**) CT examination with IV contrast. Fluid filled and air filled small bowel loops. The fluid filled loop appears hypoattenuating due to vascular parietal damage, and perivisceral fat stranding is present; findings were indicative of decompensated SBO. (**c**) At surgery, closed loops of SBO due to a bridle was evident. Vascular compromise was recognized by bluish discoloration of intestinal wall, loss of arterial pulsation, subserosal and mesenteric hemorrhage and a lack of peristalsis. After release of adhesions, bluish discoloration persisted and blocked and damaged sections of the bowel were removed.

CT confirmed the ultrasound findings in 40/43 patients. Among them, CT confirmed the positive ultrasound diagnosis of SBO in 24/43 patients, and confirmed the ultrasound negative diagnosis of SBO in 16/43 patients. In 24 out of 43 patients with SBO, CT diagnosed 10 patients with a simple ileus, 12 with a complicated ileus (Figure 2a,b) and 2 with a decompensated ileus (Figure 3a–c). In one patient with a positive ultrasound misdiagnosis of SBO, CT showed a diffuse dilatation of small bowel loops (maximum diameter 20 mm), with homogeneous parietal thickening, free fluid in the abdomen and mesenteric nodes enlargement, suspicious for diffuse enterocolitis; the patient was hospitalized and diagnosed with tuberculosis enterocolitis and was treated with medical therapy. In two patients with a negative ultrasound misdiagnosis of SBO, CT findings revealed a simple SBO. Ten patients with simple SBO obstruction were treated conservatively with complete resolution of symptoms. Among 12 patients with complicated ileus, 6 were treated conservatively. Five patients underwent surgery after 24 h of conservative management because of the worsening of laboratory values with lysis of adhesions, and freeing up and recovery of the trapped bowel. In one patient with complicated ileus, CT revealed a peritoneal carcinomatosis with bowel loops distended, omental nodes and fluid in the peritoneal cavity; the patient underwent surgery for debulking. Two patients with decompensated ileus and CT signs of vascular parietal damage were taken to the operating room and underwent bowel resection.

Ultrasound excluded the diagnosis of SBO in 14 patients, and 2 patients of them had a CT positive diagnosis of simple ileus and were considered false negative. 

CT confirmed the ultrasound diagnosis of small bowel obstruction in 24/25 patients. Among them, 12/25 presented a complicated ileus and 2/25 a decompensated ileus (Figure 3a,b). Among 12 patients with complicated ileus, 6 were treated conservatively with complete resolution of symptoms, and 5 patients underwent surgery after 24 h of conservative management because of the worsening of laboratory values. In 1 out of 12 patients with complicated ileus, CT revealed a peritoneal carcinomatosis with bowel loops distended, omental nodes and fluid in the peritoneal cavity, so the patient underwent surgery for debulking. 

Patients with decompensated ileus and CT signs of vascular parietal damage (12/25) were taken to the operating room.

In the one patient with false positive diagnosis of SBO, CT imaging revealed a diffuse enterocolitis with dilated both colonic and small bowel loops, and with parietal thickening that was subsequently diagnosed as peritoneal tuberculosis and treated with medical therapy. 

Our data demonstrated that ultrasound compared to CT imaging had a sensitivity of 92.31% (95% CI, 74.87% to 99.05%, +LR 15.69) and a specificity of 94.12% (95% CI, 71.31% to 99.85%, −LR 0.08), with a PPV (positive predictive value) of 96% and an NPV (negative predictive value) of 88.89% in the diagnosis of SBO (Table 2). Twenty-six patients (16 F/10 M), who did not undergo CT were excluded from primary analysis. Fourteen patients were hospitalized and 8 patients underwent surgery; 14 out of 26 patients (8 F/6M) had an ultrasound diagnosis of simple SBO, so they underwent immediately conservatively treatment and were admitted to the Surgical Department; 8 patients received an US diagnosis of mechanical SBO caused by incarcerated hernia (crural hernia in 7 female patients, and inguinal in 1 male patient), and they underwent surgery. In all of them, the prompt diagnosis and cause of mechanical SBO allowed rapid and minimally invasive surgery with complete recovery of the bowel loops incarcerated. Two patients were excluded because of indeterminate analysis, and indeterminate scans were defined as the presence of dilated bowel loops or abnormal peristalsis but not both. Those patients had abdominal carcinosis with decreased or absent bowel peristalsis with parietal thickening at US examination, but not dilated bowel loops. 

## 3. Discussion

SBO is a dynamic pathology. Nowadays, SBO treatment strictly depends on bowel parietal involvement. In SBO the diagnostic imaging modalities should define the presence or absence of SBO, parietal involvement and the cause and the level of SBO. CT remains the gold standard imaging modality. CT is associated with increased radiation exposure, increased cost and delayed time to diagnosis. Moreover, CT diagnosis of SBO is limited by the need to find the transition point between dilated bowel loops and decompressed bowel loops [14,21,29,32]. In 2017, Gottlieb et al. [17] published a systemic review and meta-analysis of the use of ultrasound to evaluate SBO. The authors identified 11 studies with 1178 patients—the providers who performed the ultrasounds included emergency physicians, surgeons and radiologists. In this systematic review, ultrasound was found to be 92.4% sensitive and 96.6% specific for SBO. Recent studies demonstrated that there is not a significant difference in the accuracy of detecting SBO between ultrasound and CT. Ultrasound may help the emergency physician to answer rapidly the following questions: the presence or absence of SBO, the grade of bowel involvement, the patient’s needs of emergency surgery and the clinical progress in patients treated conservatively [8,14,15,18,20,27]. Ultrasound criteria for the diagnosis of SBO are related to morphological and functional findings [27].

The obstacle to the progression of intestinal contents and fluids determines the dilatation and fluid filled bowel loops with hyperechoic spots of gas moving within the fluid, which represents the first sonographic sign evaluated in the presence of dilated loops. For those physio-pathological correlations, peristalsis is defined at ultrasound as present and/or hyperkinetic, decreased or absent. In the initial phase of SBO, the dilated loops close to the point of obstruction may be hyperperistaltic because the bowel is contracting more trying to get over the obstruction; at this stage, bowel layers and valvulae conniventes may be clearly visible but not thickened. Later with the persistent obstruction, vascular damage progresses causing a bowel layer, valvulae conniventes are thickened and bowel peristalsis is decreased (complicate ileus) or completely absent (decompensated ileus). Abnormal peristalsis, although highly suggestive, has a relatively low specificity, and must be supported by other signs [7,26,27,33,34]. In our study, the false positive case was misdiagnosed on ultrasound for the presence of free fluid and abnormal cinesis (decreased or “back and forth” movements) in absence of significantly dilated bowel loops, and in this patient, the altered peristalsis was due to diffuse peritoneal pathology (tubercular peritonitis). In the diagnosis of SBO, the most important sonographic sign is the presence of dilated bowel loops, otherwise, the diagnosis should not be questioned, and the diagnosis cannot be made based only on peristalsis activity [33]. This study demonstrated that ultrasound compared to CT imaging had a sensitivity of 92.31% (95% CI, 74.87% to 99.05%, +LR 15.69) and a specificity of 94.12% (95% CI, 71.31% to 99.85%, −LR 0.08) in the diagnosis of SBO, confirming what was previously published in other studies.

There are several limitations to our study. The sample size of our study population was small and our data were obtained through a retrospective chart review with a single-center cohort. Almost all patients with and ultrasound diagnosis of simple ileus did not undergo CT, and were directly admitted to the surgical department and/or treated conservatively. The ultrasound diagnosis and staging of SBO was made based on qualitative criteria in the ER setting.

## 4. Conclusions

Ultrasound can accurately diagnose SBO, determining the presence or the absence of pathology, with no significant difference with CT or limiting the need of CT scan in the Emergency Department [29], speeding up the patient’s management who are expeditiously admitted to the hospital. The majority of stable patients without sonographic signs of parietal damage and with present bowel kinesis are nowadays conservatively managed, and can be monitored with series ultrasound examinations that are safe and can be used to evaluate the progress of intestinal obstruction.

## Figures and Tables

**Table 1:** Ultrasound criteria for SBO diagnosis.

	Simple	Complicated	Decompensated
Bowel loops diameter	Increased	Increased	Increased
Parietal thickness	Normal	Normal or increased	Increased
Valvulae conniventes	Not thickened	Not thickened	Thickened
Peristalsis	Present and/or hyperkinetic	Decreased	Absent
Free fluid	Absent	Present	Present

**Table 2:** Performance characteristic of US for SBO compared to abdominal CT.

Total	TP	TN	FP	FN	Sensitivity (95% CI)	Specificity (95% CI)	LR+ (95% CI)	LR− (95%CI)	PPV	NPV
43	24	16	1	2	92.31% (74.87–99.05%)	94.12% (71.31–99.85%)	15.69 (2.34–105.41)	0.08 (00.2–0.31)	96%	88.89%
